# Flavan-3-ols and Vascular Health: Clinical Evidence and Mechanisms of Action

**DOI:** 10.3390/nu16152471

**Published:** 2024-07-30

**Authors:** Justyna Godos, Giovanni Luca Romano, Samuele Laudani, Lucia Gozzo, Ida Guerrera, Irma Dominguez Azpíroz, Raquel Martínez Diaz, José L. Quiles, Maurizio Battino, Filippo Drago, Francesca Giampieri, Fabio Galvano, Giuseppe Grosso

**Affiliations:** 1 Department of Biomedical and Biotechnological Sciences, University of Catania, 95123 Catania, Italy; 2 Department of Medicine and Surgery, University of Enna “Kore”, 94100 Enna, Italy; 3 Clinical Pharmacology Unit/Regional Pharmacovigilance Centre, Azienda Ospedaliero Universitaria Policlinico “G. Rodolico-S. Marco”, 95123 Catania, Italy; 4 Research Group on Food, Nutritional Biochemistry and Health, Universidad Europea del Atlántico, Isabel Torres 21, 39011 Santander, Spain; 5 Research Group on Food, Nutritional Biochemistry and Health, Universidade Internacional do Cuanza, Cuito EN250, Angola; 6 Research Group on Food, Nutritional Biochemistry and Health, Universidad de La Romana, La Romana 22000, Dominican Republic; 7 Research Group on Food, Nutritional Biochemistry and Health, Universidad Internacional Iberoamericana, Campeche 24560, Mexico; 8 Research Group on Food, Nutritional Biochemistry and Health, Universidad Internacional Iberoamericana, Arecibo, PR 00613, USA; 9 Department of Physiology, Institute of Nutrition and Food Technology “José Mataix Verdú”, Biomedical Research Centre, University of Granada, 18016 Armilla, Spain; 10 Research and Development Functional Food Centre (CIDAF), Health Science Technological Park, Avenida del Conocimiento 37, 18016 Granada, Spain; 11 Department of Clinical Sciences, Università Politecnica delle Marche, 60131 Ancona, Italy; 12 International Joint Research Laboratory of Intelligent Agriculture and Agri-Products Processing, Jiangsu University, Zhenjiang 212013, China; 13 Center for Human Nutrition and Mediterranean Foods (NUTREA), University of Catania, 95123 Catania, Italy

**Keywords:** flavan-3-ols, polyphenols, inflammation, vascular, gut microbiota

## Abstract

Cardiovascular diseases (CVDs) are one of the main causes of mortality and morbidity worldwide. A healthy diet rich in plant-derived compounds such as (poly)phenols appears to have a key role in improving cardiovascular health. Flavan-3-ols represent a subclass of (poly)phenols of great interest for their possible health benefits. In this review, we summarized the results of clinical studies on vascular outcomes of flavan-3-ol supplementation and we focused on the role of the microbiota in CVD. Clinical trials included in this review showed that supplementation with flavan-3-ols mostly derived from cocoa products significantly reduces blood pressure and improves endothelial function. Studies on catechins from green tea demonstrated better results when involving healthy individuals. From a mechanistic point of view, emerging evidence suggests that microbial metabolites may play a role in the observed effects. Their function extends beyond the previous belief of ROS scavenging activity and encompasses a direct impact on gene expression and protein function. Although flavan-3-ols appear to have effects on cardiovascular health, further studies are needed to clarify and confirm these potential benefits and the rising evidence of the potential involvement of the microbiota.

## 1. Introduction

Cardiovascular diseases (CVDs) are among the leading causes of death globally [1]. Different risk factors are involved in CVD development, such as age, high blood pressure, smoking, dyslipidemia, and diet [2]. The latest estimates from the Global Burden of Disease study reported that dietary risks represent the leading factors in affecting CVD burden [3], with an estimated 10 million CVD deaths in 2017 due to diets low in fruits, vegetables, whole grains, nuts, and seeds, while high in sodium [4]. Mechanistically, the processes leading to vascular and endothelial function consist of the activation of different pathways involved in inflammatory responses (including the nuclear factor-kappa B (NF-κB), p38, protein kinase B (PKB/Akt), and extracellular signal-regulated kinases (ERK)1/2) which impair nitric oxide (NO) synthesis and the expression of different adhesion molecules (i.e., intercellular adhesion molecule-1 (ICAM-1) and vascular cell adhesion protein-1 (VCAM-1)) [5]. Chronic protraction of such pro-inflammatory insults, especially when promoted through a suboptimal diet, may lead to substantial impairment of vascular function, the establishment of hypertension, and ultimately an increased risk of CVD [6].

There is consistent evidence that plant-based dietary patterns are associated with a reduced risk of CVD [7,8]. While adherence to a healthy diet is ideal for reducing health risks, an increasing body of research suggests the key role played by bioactive compounds [9,10]. Among the most studied, (poly)phenols achieved great interest for their beneficial effects on cardiovascular health [11]. Flavanols (also known as flavan-3-ols) represent one of the most abundant polyphenols in the human diet, mainly found in fruits, green tea, red wine, and cocoa [12]. Chemically, flavan-3-ols represent a complex subclass of flavonoids that include a variety of monomeric, oligomeric, and polymeric compounds. The main monomeric forms are (+)-catechin, (−)-epicatechin, (+)-gallocatechin, (−)-epigallocatechin, (−)-epicatechin-3-*O*-gallate, and (−)-epigallocatechin-3-*O*-gallate. Oligomers or polymers of flavan-3-ols are known as proanthocyanidins, while polymers composed exclusively of epicatechin or catechin are named procyanidins. Previous evidence agreed that flavan-3-ols might exert beneficial effects toward the vascular system [13,14], by regulating various cellular signaling pathways [15]. Additionally, epidemiological studies showed an association between higher flavan-3-ol intake and a lower risk of CVD and mortality [16,17].

An intriguing hypothesis recently investigated over the last decade involves the role of gut microbiota in mediating, at least in part, the positive effects of dietary (poly)phenols on human health [18]. The gut microbiota consists of bacteria, viruses, fungi, and eukarya which together contribute to the healthy/unhealthy state of the host [19]. Flavan-3-ols introduced by diet mainly in the form of aglycone are not completely absorbed as such but rather metabolized by the microbiota residing in the gut [20], affecting gut microbiota population and gut homeostasis [21]. A recent study highlighted intra- and inter-source differences in flavan-3-ol bioavailability, suggesting the role of substantial individual differences when considering the absorption and bioavailability of such compounds [22]. This raised a growing interest in the interindividual diversity of microbiota composition that leads to differences in the microbial-derived metabolites with great impact on health outcomes [23]. The aim of this review is to provide an overview of the results coming from the clinical trials found in the literature on flavan-3-ols and their effects on vascular health and the potential role of the gut microbiota. Moreover, we will discuss the main molecular mechanisms of flavan-3-ols and their microbial-derived metabolites.

## 2. Gut Microbiota and Cardiovascular Health

The gut microbiota consists of the totality of microorganisms cohabiting the digestive tract including bacteria, fungi, archaea, viruses, and protozoa, which communicate bidirectionally with the host through different routes including the vagus nerve, the immune system, and different microbial-derived metabolites [24,25]. The composition of the microbiota can be influenced by different factors like drug administration, stress, mode of delivery, and diet [25,26]. Gut microbiota alterations, namely dysbiosis, can lead to impaired intestinal barrier integrity (leaky gut) leading to the translocation of bacteria products into the host circulation system and the onset of an inflammatory state which have been correlated with an augmented incidence of different pathologies including cardiovascular disease [27]. Many studies investigated microbiota composition and its correlation with CVD. Comparing the gut microbiota of atherosclerotic patients with a control group, an increase in *Escherichia coli*, *Klebsiella* spp., *Enterobacter aerogenes*, and *Ruminococcus gnavus* was observed, along with a reduction in different short-chain fatty acid producers including *Roseburia intestinalis* and *Faecalibacterium prausnitzii* [28]. Similar results have been observed in works conducted by other authors in which when comparing the microbiota of patients with chronic heart failure and control participants, they demonstrated, even in this case, a significant reduction in *Faecalibacterium prausnitzii* and an increase in *Ruminococcus gnavus* [29]. In another study, the microbiota of pre-hypertensive and hypertensive patients was characterized by a reduced richness and diversity compared to the control group. These alterations were accompanied by an increase in the genera Klebsiella and *Prevotella* and a reduced abundance of beneficial bacteria including Oscillibacter, *Faecalibacterium*, Bifidobacterium, *Roseburia*, Butyrivibrio, and *Coprococcus*. Fecal transplantation from patients to germ-free mice led to an increase in blood pressure compared to control-recipient mice [30]. The establishment of a dysbiosis state has been associated with altered production of microbial-derived metabolites, which could exert detrimental effects on host health. Among the different metabolites produced by the gut microbiota, trimethylamine N-oxide (TMAO) has been largely associated with CVD [31]. TMAO derives from the liver oxidation of trimethylamine (TMA), a metabolite produced by the metabolism of foods containing l-carnitine and choline, mainly contained in animal-derived products [31]. Increased levels of TMAO were correlated with augmented cholesterol deposition and a higher risk of atherosclerosis [32]. In addition to TMAO, a longitudinal study demonstrated the association of other microbial-derived metabolites, such as p-cresol, indoxyl sulfate, and phenylacetylglutamine, with the incidence of major adverse cardiovascular events and poor survival risk [33]. Diet represents an easier way to modulate microbiota composition. Flavan-3-ol intake was demonstrated to possess prebiotic and antimicrobial properties resulting in an increased abundance of beneficial bacteria, such as *Lactobacillus*, and *Bifidobacterium,* and a reduction in *Clostridium perfringens* [34]. The microbiota changes induced by flavan-3-ol intake were demonstrated to have beneficial effects on microbial-derived metabolite production, leading to a reduction in TMAO plasmatic levels [35]. Moreover, flavan-3-ols promote beneficial effects on intestinal barrier function through the inhibition of inflammatory signals, reduced barrier permeability, and immune modulation [36].

## 3. Clinical Evidence of Flavan-3-ols on Vascular Outcomes

### 3.1. An Overview of Randomized Clinical Trials Evaluating the Effect of Flavan-3-ol Intake on Vascular Health

Flavan-3-ols are derivatives of flavans that include a variety of compounds commonly found in several plant foods, such as cocoa products, tea, apples, pears, and berries [37]. A summary of RCTs providing flavan-3-ol doses for the intervention on endothelial and vascular outcomes is presented in Table 1.

Only an early, double-blind, RCT examined the consequences of the ingestion of cocoa flavan-3-ol and procyanidin tablets, containing 234 mg flavan-3-ols and procyanidins, or placebo tablets on the platelet function of 32 healthy individuals. At the end of the trial, a decrease in P-selectin (*p* = 0.06) and adenosine diphosphate (ADP)-induced aggregation (*p* = 0.042) was observed between groups [38]. Further studies have been conducted using cocoa products. Among the studies conducted on healthy adults, a double-blind, RCT conducted on 21 healthy adults (21–55 y) investigated the effects of high-flavonoid (213 mg procyanidins and 46 mg of epicatechin) or low-flavonoid dark chocolate bar intake (46 g/day) showing an improvement in the flow-mediated dilation (FMD) of the brachial artery (1.3 ± 0.7%; *p* = 0.024) in high-flavonoid chocolate consumers likened to the control group after 2 weeks of treatment, while no differences were observed in blood pressure (BP) in both groups [39]. Similarly, in a crossover RCT conducted on 28 healthy male adults, aged between 18 and 20 years, the effects of flavan-3-ol-containing milk chocolate (providing 168 mg flavan-3-ols, 39 mg epicatechin, and catechin and 126 mg of procyanidins) was evaluated compared to white chocolate with a negligible amount of flavan-3-ol, which crossed over after 14 days: the intervention resulted in a reduction in systolic blood pressure (SBP), mean BP (from 89 ± 2 to 84 ± 2 mmHg, *p* = 0.008), and diastolic blood pressure (DBP) (from 72 ± 2 to 67 ± 2 mmHg, *p* = 0.01), while no differences were observed for white chocolate consumption [40]. Another single-blind, parallel RCT involving 44 participants (56–73 y) investigated the effects of dark chocolate supplementation, containing 30 mg of polyphenols, or polyphenol-free white chocolate. At the end of the treatment (18 weeks) a reduction in SBP (−2.9 ± 1.6 mmHg, *p* < 0.001) and DBP (−1.9 ± 1.0 mmHg, *p* < 0.001) from baseline in the intervention group was observed [44]. Similarly, an RCT conducted on 89 healthy females (18–25 y) administered with either (i) 100 g of dark chocolate (providing 3.99 catechin, 4.01 epicatechin, and 1.89 gallic acid) daily, (ii) 90 g/day of white chocolate (containing no polyphenols), or (iii) no chocolate, showed that only dark chocolate supplementation was associated with a lowering in SBP (from 115.9 ± 12.6 to 107 ± 8.6 mmHg; *p* < 0.05) and DBP (from 73 ± 9.9 to 67.7 ± 9.7 mmHg; *p* < 0.05) [45]. Another study including 39 healthy men (23–40 y) evaluated the effects of flavonoid-rich dark chocolate (providing 500 mg of catechin, epicatechin, and procyanidin) compared to white chocolate consumption on coronary flow velocity reserve (CFVR) measured by noninvasive transthoracic doppler echocardiography. The results demonstrated an increase in CFVR in the treated group (from 3.38 ± 0.49 to 4.28 ± 0.85, *p* < 0.01) likened to the non-flavonoid group [50]. In a crossover, double-blind RCT, the effect of daily consumption of 22 g/day sugar-free cocoa, 22 g/day sugar-sweetened cocoa (providing 21 mg catechin, 48 mg epicatechin, 92 mg procyanidin dimer, 98 mg procyanidin trimer, 31 mg procyanidin tetramer, 55 mg procyanidin pentamer, and hexamer, 805 mg total procyanidin) or placebo (no cocoa with 100 g/day of sugar) beverages for 6 weeks on 37 healthy participants was tested. Results displayed a protective effect of cocoa ingestion, independent of sugar content, on endothelial function measured as FMD (2.4%, 95% CI: 1.5%, 3.2% and 1.5%, 95% CI: 0.6%, 2.4%, respectively), which was increased compared to the placebo group (*p* < 0.01), while no differences were observed concerning BP in any groups [52]. Another crossover, single-blind RCT compared the effect of 20 g of polyphenol-rich dark chocolate (providing 18.99 mg epicatechin and catechin) on 42 women volunteers (21 with a normal BMI, 13 overweight, and 8 obese). The results showed a decreased SBP (*p* = 0.007) and DBP (*p* = 0.003) after 4 weeks of polyphenol-rich dark chocolate administration compared to pretreatment [53]. In another double-blind, parallel-arm RCT, 90 elderly individuals consumed a cocoa drink containing 990 mg (high flavan-3-ols), 520 mg (intermediate flavan-3-ols), or 45 mg (low flavan-3-ols) of flavan-3-ols once daily for 8 weeks. Results displayed a reduction in SBP (high cocoa flavanol (CF) from 141.1 to 131 mmHg, intermediate CF from 142.4 to 134.2 mmHg, *p* < 0.0001) and DBP (high CF from 84.5 to 79.7 mmHg, intermediate CF from 86.4 to 83 mmHg, *p* < 0.0001) after 8 weeks of treatment, as well as a notable decrease in SBP (*p* = 0.0018) and DBP (*p* = 0.007) comparing high and intermediate CF versus low CF [54]. In another double-masked, parallel-group RCT, the effect of flavan-3-ol intake in 22 young (<35 y) and 20 elderly (50–80 y) healthy males allocated to consume a cocoa-flavan-3-ol drink (providing 900 mg of flavan-3-ols, 128 mg (−)-epicatechin, 14 mg (−)-catechin, 4 mg (+)-catechin, and 754 mg dimers–decamers) or a nutrient-matched, cocoa-flavan-3-ol-free control drink was evaluated. The results displayed an improvement in FMD (young: 7.8 ± 0.2% and elderly: 6.3 ± 0.3%; *p* < 0.05), reduced pulse wave velocity (PWV) (in young: from 6.0 ± 0.1 to 5.6 ± 0.1 m/s; in elderly: from 9.3 ± 0.5 to 8.5 ± 0.4 m/s; *p* < 0.05) and total peripheral resistance, as well as increased arteriolar and microvascular vasodilatory capacity and central DBP (*p* < 0.05) in both groups after 2 weeks, with a decreased aortic augmentation index (AIX) and SBP (*p* < 0.05) in elderly participants [63]. In a parallel-arm, double-blind RCT conducted on 90 elderly participants (mean age 70 y) without any significant clinical medical conditions, the effects of high-, intermediate-, or low-flavan-3-ol drinks (providing 993 mg total flavan-3-ols, 520 mg total flavan-3-ols, or 48 mg total flavan-3-ols, respectively) for 8 weeks were evaluated, resulting in a reduction in SBP in participants after treatment with high (from 138 ± 1.28 to 130 ± 1.21 mmHg; *p* < 0.0001) and intermediate (from 137 ± 1.37 to 130.2 ± 1.28 mmHg; *p* < 0.0001) flavan-3-ol cocoa compared with the low-treated group (*p* < 0.05), while DBP was reduced in all three groups without any significant changes between them [66]. Another double-blind, parallel-group RCT, involving 100 healthy individuals aged between 35 and 60 years and divided into two groups to consume a cocoa-flavan-3-ol-containing drink (providing 900 mg total flavan-3-ols, 146 mg monomers, 128 mg (−)-epicatechin, 14 mg (−)-catechin, 4 mg (+)-catechin, and 754 mg dimers–decamers) or a placebo, for 1 month, showed an increase in FMD (1.2, 95% CI: 1.0, 1.4%) and a decrease in SBP (−4.4, 95% CI: −7.9, −0.9 mmHg) and DBP (−3.9, 95% CI: −6.7, −1.1 mmHg) and PWV (−0.4, 95% CI: −0.8, −0.04) in the intervention group [70].

Various studies, conducted in hypertensive individuals and high-CVD-risk individuals, resulted in promising results as well. A crossover RCT explored the impacts of dark chocolate consumption in 20 never-treated, grade I essential hypertensive individuals (mean age 43 y) and normotensive control individuals (mean age 34 y) receiving 100 g/day of dark chocolate (containing 88 mg of flavan-3-ols, 21.91 mg catechin, 65.97 mg epicatechin, 0.59 mg quercetin, 0.03 mg kaempferol, and 0.31 mg isorhamnetin) or 80 g of white chocolate. The treatment led to a reduction in SBP (−11.0 ± 6.3 mmHg; *p* = 0.0001) and DBP (−6.2 ± 4.2 mmHg; *p* = 0.0001) in comparison to the baseline. A response of SBP and DBP to treatment was observed also in the control group, with a significant effect of treatment (*p* < 0.0001) and time (*p* < 0.0001). Furthermore, an augmentation of FMD likened to the baseline and to the white chocolate group was observed in both the hypertensive and control groups (*p* < 0.0001) [41]. In a double-blind RCT, 32 postmenopausal, hypercholesterolemic women were recruited to evaluate the effects of 6-week supplementation with a high-flavan-3-ol cocoa beverage, containing 446 mg of flavan-3-ols, or a low-flavan-3-ol cocoa beverage, containing 43 mg of flavan-3-ols: at the end of the study, brachial artery hyperemic blood flow increased by 76% after the high-flavan-3-ol treatment (*p* < 0.05) compared to the baseline, but a decrease in SBP and DBP by 9.3% and 6.5%, respectively (*p* < 0.05) in the low-flavan-3-ol group compared to the high-flavan-3-ol group was reported [42]. Another crossover RCT was conducted on 19 essential hypertensive participants with impaired glucose tolerance (mean age of 44 y) receiving either 100 g/day of chocolate rich in flavan-3-ols (providing 110.9 mg epicatechin, 36.12 mg catechin, 2.5 mg quercetin, 0.03 mg kaempferol, and 0.2 mg isorhamnetin) or flavan-3-ol-free white chocolate for 15 days followed by washout and crossover. The results showed that flavan-3-ol-rich chocolate was able to reduce both SBP (−3.82 ± 2.40 mmHg) and DBP (−3.92 ± 1.98 mmHg) and increase FMD (*p* < 0.0001) while no effects were observed for the flavan-3-ol-free chocolate [48]. A double-blind, crossover RCT investigated the effects of cocoa drink supplementation (150 mL twice a day, 900 mg flavan-3-ol/day) on 20 participants with essential hypertension for 2 weeks of treatment (followed by 1 week of washout before crossover), resulting in an increase in insulin-stimulated changes in brachial artery diameter compared to the placebo group (8.1 ± 1.2 versus 5.0 ± 1.3; *p* = 0.028), but was not able to significantly reduce BP [49]. A double-blind, parallel RCT, was conducted on 49 overweight/obese participants, randomly assigned to intake high-flavan-3-ol (902 mg flavan-3-ol) or low-flavan-3-ol (36 mg flavan-3-ols) cocoa, in combination or not with exercise for 12 weeks. Compared to the low-flavan-3-ol group, the high-flavan-3-ol group showed an increase in FMD chronically (over 12 weeks) by 1.6% (*p* < 0.01) and a reduction in DBP by 1.6 mmHg and mean arterial BP by 1.2 mmHg (*p* < 0.05), independent of exercise [47]. Another double-blind, crossover RCT, involving 16 patients with coronary artery disease (mean age 64 y) receiving dietary high-flavan-3-ol intervention (providing 750 mg flavan-3-ols) and low-flavan-3-ol intervention for 30 days showed an improvement in brachial artery FMD by 4.6% (*p* < 0.05) and a reduction in SBP (−4.2 ± 2.7 mmHg; *p* = 0.013) in the treated group compared to the control group [51]. A double-blind, RCT conducted on 20 participants with congestive heart failure evaluated the effects of 40 g of flavan-3-ol-rich chocolate (providing 10.8 mg catechin, 36 mg epicatechin) or 28.4 g of control chocolate. The treatment led to an improvement in FMD after 4 weeks of daily intake of flavan-3-ol-rich chocolate (from 5.15 ± 1.23 mm to 5.25 ± 1.22 mm; *p* = 0.02), while no differences were observed in BP and heart rate in either group [55]. Finally, a study conducted on another category of patients (hemodialyzed), including 57 participants supplemented with chocolate-flavan-3-ol rich beverages (900 mg of flavan-3-ols per day) or a placebo for 30 days, showed an increase in FMD (from 3.4 ± 0.9% to 3.9 ± 0.8% vs. placebo, from 3.5 ± 0.7% to 3.5 ± 0.7%; *p* < 0.001), a reduction in DBP (from 73 ± 12 to 69 ± 11 mmHg vs. placebo, from 70 ± 11 to 73 ± 13 mmHg; *p* = 0.03) and an increase in heart rate (from 70 ± 12 to 74 ± 13 bpm vs. placebo, from 75 ± 15 to 74 ± 13 bpm; *p* = 0.01) [68].

In contrast, some studies did not detect substantial differences after supplementation. A crossover, double-blind, RCT investigated the effects of 70 g/day of flavan-3-ol-enriched chocolate consumption (providing 1078 mg flavan-3-ols, 349 mg epicatechins) or normal-flavan-3-ol chocolate on increased endothelium-dependent vasodilatation in 41 healthy overweight men (45–70 y) leading to an increase in FMD (*p* = 0.01) and the AIX (*p* = 0.024) by about 1% as well as soluble ICAM (sICAM) 1, sICAM3, and leukocyte adhesion markers (*p* < 0.05), independent of the type of chocolate consumed [58]. In another parallel-arm RCT, 60 overweight or obese premenopausal women (25–45 y) were instructed to consume dark chocolate snacks along with a once-daily sugar-free cocoa beverage (providing 270 mg of flavan-3-ols) or non-chocolate snacks plus a sugar-free non-cocoa beverage for 18 weeks: results showed that both groups experienced reductions in SBP and DBP with no significant changes between groups [60]. Among other studies with contrasting results, an RCT investigated the effects of 10 g or 5 g of cocoa consumption (providing 261.8 mg flavan-3-ols, 45.8 mg epicatechin, 130.9 mg flavonols, and 22.9 mg epicatechin, respectively) on BP and endothelial function n 101 adults with stage 1 hypertension for 8 weeks: daily cocoa intake did not ameliorate BP and endothelial function but led to BP reduction in a subgroup of participants in angiotensin-converting enzyme inhibitors (24 h SBP: −3.2 ± 9.3 vs. 3.6 ± 8.5; *p* = 0.038, 24 h DBP: −2.0 ± 5.0 vs. 2.3 ± 5.6 mm Hg; *p* = 0.023) whereas daily consumption of 5 g of cocoa-containing products reduced SBP (−4.6 ± 3.2 vs. 1.8 ± 2.8 mm Hg; *p* = 0.009) and ameliorated endothelial activity (3.1 ± 2.3 vs. −3.4 ± 7.4%; *p* = 0.031) [71]. Also, a placebo-controlled, crossover RCT was conducted in 30 middle-aged individuals who were overweight and moderately obese to evaluate the effect of 37 g/day dark chocolate and a sugar-free cocoa beverage (total flavan-3-ols = 814 mg/day) consumption against a low-flavan-3-ol chocolate bar and a beverage mix without cocoa and no added sugar (total flavan-3-ols = 3 mg/day) on arterial stiffness and endothelial function. After 4 weeks of treatment, no changes were observed in fasting BP or heart rate, but some positive acute responses after the last intervention administration were seen [i.e., augmentation in the diameter of the brachial artery before reactive hyperemia (*p* = 0.001) and at the peak dilation following cuff release (*p* = 0.0001), as well as an increase in resting (*p* = 0.04) and peak (*p* = 0.03) hyperemic blood flow, and increases in SBP (*p* = 0.02), DBP (*p* = 0.005) and heart rate (*p* = 0.02)] compared to the control treatment [62]. Moreover, some other studies showed no important changes after supplementation. In an RCT in which 25 male subjects (mean age 38 y), were supplemented with 26 g/day of cocoa powder and 12 g/day of sugar (providing 98 mg epicatechins, 35 mg catechins, 41 mg procyanidin B2, and 24 mg procyanidin C1) or only with 12 g/day of sugar for 12 weeks, did not show significant effects on BP in both groups [43]. Similarly, a double-blind, fixed-dose, parallel-group RCT involving 90 healthy participants, showed that dark chocolate and cocoa (providing 754.71 mg total proanthocyanidins) were not associated with beneficial effects on BP. However, dark chocolate and cocoa groups displayed an increased pulse rate at the midpoint and at the end of treatment [46]. An RCT investigated the effects of 20 g/day of flavan-3-ol-rich chocolate (providing 400 mg of flavan-3-ol) or low-flavan-3-ol chocolate (containing < 60 mg of flavan-3-ol) on BP and endothelial function in 42 healthy pregnant women. No differences neither on FMD or on BP were observed [56]. In another study designed as a double-blind, parallel RCT and involving 143 healthy participants, the effects of the intake of a 200 mL/day cocoa drink, a 200 mL/day theobromine drink, or a 200 mL/day theobromine + cocoa drink (providing 325 mg flavan-3-ols, 150 mg theobromine, 850 mg theobromine, 325 mg flavan-3-ols, and 1000 mg theobromine, respectively) for 4 weeks were evaluated, without reporting significant differences for SBP, DBP, and HR [57]. In a double-blind, placebo-controlled, parallel RCT involving 47 healthy participants, supplementation with 1.4 g of cocoa extract (providing 414.26 mg total flavan-3-ols, 153.44 mg epicatechin, 14.56 mg catechin, 99.40 mg dimer B2, 13.44 mg dimer B1, and 133.53 mg oligomeric procyanidins) or controlled meals for 4 weeks led to an improvement in BP and a decrease in sICAM-1 in both groups [59]. In a crossover RCT, 24 normocholesterolemic and 20 moderately hypercholesterolemic volunteers received 30 g/day of a cocoa product rich in polyphenols (providing 416.4 mg of total polyphenols, 44,1 mg flavan-3-ols, 9.3 mg epicatechins, 18 mg catechins, and 16.5 mg procyanidin B2) plus milk or a control, consisting of milk. At the end of the treatment period, no significant changes were reported in BP and heart rate among the groups [61]. A double-blind, parallel RCT evaluated the effects of an acute and subchronic daily intake of cocoa tablets for 4 weeks, supplementing 250 mg/day of catechin in 38 young healthy participants aged between 18 and 40 years. Neither in acute nor in subchorionic administration were any changes in cardiovascular functions observed [65]. In a double-blind, crossover RCT, a total of 32 individuals (mean age 55 y) diagnosed with hypertension, were divided to be supplemented with high-flavan-3-ol dark chocolate (1064 mg flavan-3-ols/day for 6 weeks) or low-flavan-3-ol dark chocolate (88 mg flavan-3-ols/day for 6 weeks), resulting in no changes on BP, although low-flavan-3-ol dark chocolate showed a significant increase in HR [69]. In another double-blind, two-parallel-arm RCT, the influence of cocoa extract capsule supplementation (providing 2000 mg cocoa flavan-3-ols) on BP and platelet function was evaluated, but the results showed no relevant changes in SBP, DBP, and platelet function after 2 weeks of intervention [67]. In a crossover RCT involving 22 adults aged 33–64 years with mild hypertension, the effect of 49 g/day dark chocolate (providing 602.7 mg flavan-3-ols) intake on cardiovascular risk factors was evaluated. Results showed that after 8 weeks, no effects by chocolate intake on cardiovascular risk factors were observed [64]. Another RCT investigated the consequences of intaking 10 g of cocoa-rich chocolate (providing 10.4 mg catechin, 14.4 mg procyanidin dimer (B2), 26.1 mg epicatechin, and 8.53 mg procyanidin trimer (C1)) for 6 months, showing no relevant changes comparing groups for SBP (*p* = 0.391), DBP (*p* = 0.622) or brachial–ankle PWV (*p* = 0.263), but a decrease in pulse pressure (PP) was observed in the intervention group (−2.05 mm Hg; 95% CI: −4.08, −0.02; *p* = 0.048) compared to the control group [72].

It is noteworthy to mention some limitations of the trials included. First, there was no consistency in terms of length, dose, type of intervention (tablets vs. food), health status of participants, and study design; thus, a formal meta-analysis of results is hard to interpret. Second, from a clinical standpoint, the formulations themselves were not univocal in terms of molecules included; hence, it should be taken into account that the effects solely attributed to simple flavan-3-ols may also depend on other components used.

### 3.2. A Summary of Randomized Clinical Trials Investigating the Impact of Catechin Supplementation on Vascular Health

Another group of studies were conducted focusing their attention on green tea catechins. Studies conducted in both healthy and unhealthy individuals resulted in contrasting results presented in Table 2.

A double-blind, parallel RCT, conducted on 111 healthy adult volunteers aged between 21 and 70 years old and administered with two capsules per day of *Camellia sinensis* (containing 400 mg decaffeinated catechin green tea extract) or two placebo capsules for 3 months, resulted in a 3 mmHg reduction in SBP (*p* < 0.05) in the treated group (from 131 ± 6.3 to 128 ± 6.3 mmHg) with no effects on DPB [73]. Another three-arm parallel, double-blind RCT was conducted on 49 healthy adult men aged 18–35 years who were administered 450 mg green tea (about 240 mg catechins) or sour tea (at least 250 mg of anthocyanin) and one placebo group consuming 450 mg of maltodextrin for 6 weeks and showed a reduction in SDP (from 124.4 ± 5.5, after 4 weeks: 114.4 ± 7.4, *p* = 0.004) and DBP (*p* = 0.007) in the sour tea group when compared with pre-intervention values, whereas no significant differences were observed in the green tea and control groups [74]. However, a double-blind, parallel RCT involving 22 healthy postmenopausal women (62–73 y) who were administered one bottle of green tea beverage with 615 mg of total catechins or a placebo beverage with 92 mg of catechins for 4 weeks showed no changes compared to the other groups [75]. Another study included 51 subjects were randomly divided to receive beverages containing high catechin (400 mg/day) or low catechin (100 mg/day) for 9 weeks, followed by 2 weeks of washout, resulting in no significant difference between the high and low catechin group in any CVD risk factors [76]. Similarly, 46 overweight women aged 19–57 years old received a low-calorie diet with three capsules of green tea, three times a day (total intake of tea catechins 1125 mg/day), or three capsules of placebo (maltodextrins) total for 3 months, and showed a decrease in SBP and DBP in both the intervention and control group, with no change and no differences in heart rate [77]. Another RCT conducted on 33 healthy male volunteers who received six capsules per day containing green tea extracts (714 mg/day of green tea polyphenols) or placebo, displayed no effects in the intervention on endothelium-dependent or independent vascular function, as well as no changes in plasma concentrations of asymmetric dimethylarginine [78]. Similar results were detected in another double-blind RCT involving 50 older adults assigned to receive 350 mL of green tea containing 630.9 mg of catechins or a beverage without catechins for 14 weeks; also in this study, no significant differences in the change between the two groups were found [79]. Moreover, an RCT involving 114 healthy subjects receiving either barley extract without catechin (placebo), a “Sunrouge” extract containing 11.2 mg anthocyanin and 323.6 mg (−)-epigallo-catechin-3-gallate (EGCG), or a “Yabukita” extract containing 322.2 mg EGCG for 12 weeks showed an increase following intake of the “Sunrouge” extract (*p* < 0.05) [80].

**Table 2:** Main characteristics of randomized clinical trials concerning catechins supplementation and cardiovascular risk factors.

Author, Year, Country	Study Design	Participants (Mean Age)	Duration	Treatment	Polyphenol Constituent (Daily Intake)	Comparison	Main Findings
Fukino, 2005, Japan [81]	Controlled	66 patients with borderline diabetes or diabetes (53 y)	2 mo	One packet of GTE/powder	544 mg polyphenols, 456 mg catechins	No intervention	No significant changes were observed between groups.
Diepvens, 2005, The Netherlands [77]	Double-blind, placebo-controlled	46 overweight women (placebo: 41 y; green tea: 41 y)	87 d	GTE capsules with low-calorie diet	1125 mg tea catechins	Maltodextrin capsules	No changes were observed between groups.
Hill, 2007, Australia [82]	Placebo-controlled	38 overweight or obese postmenopausal women (58 y)	12 wk	EGCG capsules + walking for 45 min at 75% of age-predicted maximum heart rate	300 mg EGCG	2 lactose capsules/d + walking for 45 min at 75% of age-predicted maximum heart rate 3/wk	A significant reduction in HR was observed in the EGCG group (*p* < 0.01).
Nagao, 2007, Japan [83]	Double-blind, placebo-controlled	240 women and men with visceral fat-type obesity (41 y)	12 wk	GTE beverage high in catechins	583 mg total catechins, 42.84 mg catechin, 40.12 mg catechin gallate, 127.5 mg gallocatechin, 139.74 mg gallocatechin gallate, 32.3 mg epicatechin, 30.94 mg epicatechin gallate, 60.36 mg epigallocatechin, and 100.3 mg epigallocatechin gallate	Control beverage (96 mg catechins)	Compared with the control group, in the catechin group, a greater decrease in SBP for subjects with initial SBP values of 130 mm Hg or higher was observed (*p* < 0.05).
Fukino, 2008, Japan [84]	Crossover	60 volunteers with fasting blood glucose levels of ≥6.1 mmol/L or nonfasting blood glucose levels of ≥7.8 mmol/L (52 y)	2 × 2 mo (no washout)	GTE powder	544 mg polyphenols, 456 mg catechins	No intervention	No significant changes were observed after intervention.
Frank, 2009, UK [78]	Double-blind, placebo-controlled	33 healthy men (treatment group: 41 y; control group 40 y)	3 wk	GTE capsules	714 mg of green tea polyphenols, ~670 mg flavanols	Placebo capsules (maltodextrin and caffeine)	No changes were observed after intervention.
Hsu, 2008, Taiwan [85]	Double-blind, placebo-controlled	78 obese women (green tea: 43 y; placebo: 43 y)	12 wk	Dried, powdered GTE capsules	491 mg catechins, 302 mg EGCG	Placebo capsules (cellulose)	No significant differences were observed when comparing the two groups.
Brown, 2009, UK [86]	Double-blind, placebo-controlled	88 overweight or obese male subjects (53 y)	8 wk	EGCG capsules	800 mg EGCG	Placebo capsule (lactose)	Compared to the placebo, EGCG treatment reduced DBP (*p* = 0.014).
Nagao, 2009, Japan [87]	Double-blind, placebo-controlled	43 patients with T2DM (catechin group: 64 y; control group 62 y)	12 wk	Catechin-rich beverage	582.8 mg catechins	Control beverage with 96.3 mg catechins	No significant differences were observed between groups.
Nantz, 2009, USA [73]	Double-blind, placebo-controlled	111 healthy adult volunteers (29 y)	3 mo	Camellia sinensis compounds capsules	polyphenols > 80%, catechins > 80%, EGCG > 45%	Placebo capsules (microcrystalline cellulose)	Treatment led to a significant reduction in SBP (*p* < 0.05).
Basu, 2011, USA [88]	Single-blind, placebo-controlled	35 obese subjects with MetS (42 y)	8 wk	(i) Green tea; (ii) GTE	(i) 440 mg EGCG, 220 mg EGC, 180 mg ECG, and 88 mg EC; (ii) 460 mg EGCG, 240 mg EGC, and 120 mg ECG e 50 mg EC	Water	No effects were observed after treatment.
Brown, 2011, UK [89]	Double-blind, placebo-controlled, crossover	64 overweight and obese sedentary males (placebo: 49 y; intervention: 49 y)	2 × 6 wk (2 wk washout)	DGT capsules	EGCG 40.71%, EGC 16.27%, EC 8.74%, ECG 6.02%, GC 2.02%, GCG 1.27%, catechin 1.16%, gallic acid 0.75%, and catechin gallate 0.03% in each capsule	Placebo capsules (lactose)	No effect of treatment on any of the ambulatory BP-monitoring parameters.
Hsu, 2011, Taiwan [90]	Double-blind, placebo-controlled	68 subjects with BMI > 25 kg/m2 and T2DM for more than one year (GTE: 50 y; placebo: 52 y)	16 wk	DGT capsules	856.8 mg EGCG, 236.1 mg ECG, 115.5 mg EGC, 71.9 mg EC, 63.7 mg GCG, and <1.05 mg GC	Placebo capsules (cellulose)	No statistically significant differences were observed between groups.
Sone, 2011, Japan [76]	Placebo-controlled	51 individuals (high-catechin group: 43 y; low-catechin group: 48 y)	9 wk treatment	Catechin-enriched green tea beverage	400 mg catechins	Control beverage with 100 mg catechins	No significant differences were observed between groups.
Bogdanski, 2012, Poland [91]	Double-blind, placebo-controlled	56 obese, hypertensive subjects (GTE group: 49 y; placebo group 51 y)	3 mo	GTE capsules	208 mg EGCG	Placebo capsules (microcrystalline cellulose)	In the treated group, both SBP and DBP significantly decreased (*p* < 0.01).
Suliburska, 2012, Poland [92]	Double-blind, placebo-controlled	46 obese patients (GTE group: 48 y; placebo group: 52 y)	3 mo	GTE capsules	208 mg EGCG	Placebo capsules (microcrystalline cellulose)	No significant changes in SBP and DBP.
Miyazaki, 2013, Japan [79]	Double-blind, placebo-controlled	50 older adults participating in a pedometer-based walking program (69 y)	14 wk	Green tea catechins beverage	630.9 mg total catechin, 125.7 mg GC, 114 mg EGC, 30 mg catechin, 34.7 mg EC, 143.2 mg EGCG, 112.6 mg GCG, 45.8 mg ECG, and 24.8 mg CG	Control beverage (88.7 mg total catechin)	No significant changes were shown when comparing groups.
Liu, 2014, Taiwan [93]	Double-blind, placebo-controlled	77 subjects with type 2 diabetes and lipid abnormalities (GTE group: 55 y; cellulose group: 53 y)	16 wk	GTE capsules	856.8 mg EGCG, 236.1 mg ECG, 115.5 mg EGC, 71.9 mg EC, 63.7 mg GCG, and <1.05 mg GC	Placebo capsules (cellulose)	Treatment with GTE did not show significant changes in BP.
Takahashi, 2014, Japan [75]	Double-blind, placebo-controlled	22 healthy postmenopausal women (placebo: 66 y; green tea: 66 y)	4 wk	Green tea beverage	615 mg catechins	Placebo beverage (92 mg catechins)	No differences in BP between the groups were observed.
Dower, 2015, The Netherlands [94]	Double-blind, placebo-controlled, crossover	33 healthy (pre)hypertensive men and women (SBP between 125 and 160 mmHg) (66 y)	3 × 4 wk (2 × 4 wk washout)	(i) Epicatechin capsules; (ii) quercetin capsules	(i) 100 mg epicatechin; (ii) 160 mg quercetin-3-glucoside	Placebo capsules	Epicatechin and quercetin supplementation significantly decreased soluble endothelial selectin (*p* = 0.03 and *p* = 0.03, respectively).
Chen, 2016, Taiwan [95]	Double-blind, placebo-controlled	77 women with central obesity (GTE: 44 y; placebo: 44 y)	12 wk	DGT capsules	EGCG 856.8 mg, ECG 236.1 mg, EGC 115.5 mg, EC 71.9 mg, GCG 63.7 mg, and GC < 1.05 mg	Placebo capsules (microcrystalline cellulose)	There were no significant percentage reductions in BP between groups.
Gutiérrez-Salmeàn, 2016, India [96]	Double-blind, placebo-controlled	30 subjects with hypertriglyceridemia (37 y)	4 wk	Epicatechin capsules	100 mg epicatechin	Placebo capsules	No significant changes in SBP and DBP were observed when comparing groups.
Lu and Hsu, 2016, Taiwan [97]	Double-blind, placebo-controlled	64 women with moderate or severe acne vulgaris (GTE 28 y, placebo 30 y)	4 wk	DGT capsules	EGCG 856.8 mg ECG 236.1 mg, EGC 115.5 mg, EC 71.9 mg GCG 63.7 mg, and GC < 1.05 mg	Placebo capsules (microcrystalline cellulose)	No changes were observed after intervention.
Kafeshani, 2017, Iran [74]	Double-blind, placebo-controlled	49 healthy adult men (green tea: 20 y; sour tea: 20 y; placebo: 21 y)	6 wk	(i) Green tea tablets; (ii) sour tea tables	(i) ~240 mg catechins; (ii) at least 250 mg anthocyanins	Placebo tablets (maltodextrin)	A reduction in DBP in the sour tea group compared to the baseline (*p* = 0.007) and a significant reduction in SBP (*p* = 0.004) compared to the placebo.
Saarenhovi, 2017, Finland [98]	Double-blind, placebo-controlled, crossover	57 otherwise healthy subjects with borderline hypertension (130–139/85–89 mmHg) or unmedicated mild hypertension (140–165/90–95 mmHg) (55 y)	2 × 4 wk (4 to 5 wk washout)	Epicatechin capsules	100 mg epicatechin	Placebo capsules (microcrystalline cellulose)	Treatment induced significant increase in FMD% at the first visit (*p* = 0.04), last visit (*p* = 0.02), and for both visits combined (*p* < 0.01) but not significantly compared to placebo. DBP at end of treatment was −3.3 mmHg (*p* = 0.008).
Kirch, 2018, Germany [99]	Double-blind, placebo-controlled, crossover	47 overweight or obese nonsmokers with clear signs of MetS (M: 36 y; F: 35 y)	2 × 2 wk (2 wk washout)	Epicatechin capsules	25 mg epicatechin	Placebo capsules (mannitol)	No differences were observed between treatments.
Maeda-Yamamoto, 2018, Japan [80]	Double-blind, placebo-controlled	114 healthy subjects, with an SBP value of ≤125 and <155 and a DBP value < 95, or a DBP of ≤75 mmHg and <95 mmHg and an SBP < 155 mmHg (40 y)	12 wk	(i) Green tea cultivar Sunrouge extract; (ii) green tea cultivar Yabukita extract	(i) 11.2 mg anthocyanin, 323.6 mg EGCG, 85.7 mg EGC, 12.3 mg EGCG3”Me, 40.4 mg flavonols; (ii) 322.2 mg EGCG, 413.1 mg EGC, and 54.8 mg flavonols	Barley extract without catechin	After “Sunrouge” extract treatment, participants showed increased blood pressure (*p* < 0.05).

Abbreviations: BP (blood pressure); d (day); DBP (diastolic blood pressure); DGT (decaffeinated green tea extract); EC (epicatechin); ECG (epicatechin gallate); EGC (epigallocatechin); EGCG (epigallocatechin gallate); EGCG3”Me (epigallocatechin-3-=-(3-O-methyl)-gallate; F (female); GC (Gallocatechin); GCG (Gallocatechin gallate); GTE (green tea extract); HR (heart rate); MetS (metabolic syndrome); M (male); mo (month); SBP (systolic blood pressure); T2DM (type 2 diabetes mellitus); wk (week); y (year).

Studies conducted on unhealthy individuals included mostly patients with cardiometabolic risk factors. Most studies provided interventions with green tea as a drink or an extract. A 12-week intervention study investigated the effect of green tea extract on 240 individuals (25–55 y) with visceral fat-type obesity who were administered either green tea (583 mg/day) or a control drink (96 mg catechins/day), and showed that individuals with higher baseline SBP had a better effect of the treatment compared to controls (*p* < 0.05) with no effects on DBP [83]. The same group performed a similar study on 43 patients with type 2 diabetes mellitus, administering for 12 weeks (plus a 4-week follow-up period) green tea containing either 582.8 mg of catechins or 96.3 mg of catechins (control group). Results displayed a marginal reduction in SBP in the treated group during the trial but no significant differences between the two groups [87]. In contrast, a more recent study conducted in 77 women with central obesity (20–60 y) randomly assigned to receive high-dose green tea (consumption of 856.8 mg EGCG/day) or a placebo for 12 weeks reported no reductions between groups and in other measurements analyzed [95].

Among studies on green tea extracts in obese individuals, most studies reported null findings. A double-blind RCT, conducted on 78 obese women, examined the consequence of green tea extract intake (about 500 mg of catechins/day) three times a day for 12 weeks compared to the placebo group. Results showed no significant difference between SBP and DBP in either group nor between them after the follow-up [85]. A similar RCT was conducted on 35 obese participants with metabolic syndrome (mean age 42.5 ± 1.7 y), who were randomly assigned to intake green tea (four cups/day), green tea extract (two capsules), or four cups water/day, and showed that the supplementation did not have any effect on metabolic syndrome features, including SBP (*p* = 0.94 for green tea, *p* = 0.86 for green tea extract vs. no treatment) and DBP (*p*= 0.96 for green tea, *p* = 0.48 for green tea extract vs. no treatment) [88]. Another double-blind, crossover RCT conducted on 64 sedentary males diagnosed as overweight and obese (40–69 y) who were allocated to receive decaffeinated green tea extract for 6 weeks and a crossover, did not lead to any effect on the ambulatory parameters [89]. Another study conducted on 46 slightly younger obese individuals (30–60 y) with similar intervention and duration did not display any significant differences comparing groups [92]. Finally, in another double-blind RCT, 56 obese and hypertensive subjects, aged 30–60 years old, were randomly assigned to ingest a daily dose of one capsule of 379 mg green tea extract containing 208 mg of EGCG or a matching placebo capsule of pure microcrystalline cellulose for 3 months. Results reported a reduction in both SBP (from 145 ± 10 to 141 ± 8 mmHg, *p* = 0.004) and DBP (from 88 ± 4 to 84 ± 3 mmHg, *p* < 0.001) in the treated group compared with the placebo group [91]. Also, studies on diabetic individuals led to mostly null results. Only the first study, conducted on 66 diabetic or prediabetic patients (aged 32–73 y) who received a packet of green tea extract containing 456 mg catechins for 2 months or no intervention, resulted in an improvement in both SBP (from 139.3 ± 15.7 to 131.6 ± 20.8 mmHg) and DBP (from 92.5 ± 11.1 to 83.3 ± 12,6), although with no significant differences with the control group [81]. Then, a crossover RCT was conducted in 60 volunteers (32–73 y) with fasting blood glucose levels of ≥6.1 mmol/L or nonfasting blood glucose levels of ≥7.8 mmol/L, who consumed a packet of green tea-extract powder containing 456 mg catechins daily for 2 months and a 2-month non-intervention period, and showed no significant changes in SBP (*p* = 0.43) whereas the reduction in DBP associated with the intervention was of borderline statistical significance (*p* = 0.06) [84]. A double-blind RCT aimed to examine the effect of a decaffeinated green tea extract providing a daily dose of 856 mg of epigallocatechin gallate or a placebo (cellulose) on 68 subjects with a BMI of >25 kg/m^2^ and type 2 diabetes for 16 weeks and did not display a statistically significant difference between groups for any measured variable [90]. Similarly, a study conducted on 77 patients aged between 20 and 65 years and diagnosed with type 2 diabetes mellitus and lipid abnormalities explored the effects of 16 weeks of treatment with decaffeinated green tea extract (856.8 mg of EGCG) compared with a placebo. The results did not show any significant changes in the parameters investigated [93]. The same null results were obtained by a double-blind RCT conducted on 64 women between 25 to 45 years old with moderate or severe acne supplemented with decaffeinated green tea extract, providing a daily dose of 856 mg of epigallocatechin gallate upon or a placebo (cellulose) for 4 weeks [97].

Studies using purified catechin extracts resulted in more significant, yet not fully consistent results. A study comparing the effect of 8 weeks of dietary supplementation with epicatechin (400 mg capsules twice daily) vs. placebo lactose on overweight or obese male subjects (40–65 y) resulted in a reduction in DBP (mean change: placebo −0.058 (SE 0.75) mmHg vs. EGCG −2·68 (SE 0.72) mmHg; *p* = 0.014) but no significant effects on SBP [86]. Another double-blind RCT conducted on 33 healthy prehypertensive men and women, with a mean age of 66.4 ± 7.9 years, and receiving epicatechin (100 mg/day), quercetin-3-glucoside (160 mg/day), or placebo capsules for 4 weeks showed that either epicatechin and quercetin supplementation decreased soluble endothelial selectin (−7.7 ng/mL, 95% CI: 14.5, 0.83; *p* = 0.03 and −7.4 ng/mL, 95% CI: 14.3, 0.56; *p* = 0.03, respectively), whereas all other markers of endothelial dysfunction and the derived z score for endothelial dysfunction did not change significantly [94]. Another study conducted on 38 overweight or obese postmenopausal women (45–70 y) instructed to intake two capsules containing 300 mg/day of epigallocatechin gallate or lactose (placebo) for 12 weeks resulted in a reduction in heart rate in the treated group (*p* < 0.01) although no significant differences were observed in arterial compliance, endothelial function, and BP in either treatment [82]. On the contrary, a double-blind, multicenter RCT including 30 subjects diagnosed with hypertriglyceridemia (18–50 y), instructed to take two capsules of 25 mg epicatechin or a placebo for 4 weeks twice a day (total daily dose of 100 mg) [96] and a double-blind, crossover RCT conducted on 47 overweight or obese nonsmokers with clear signs of metabolic syndrome administered with 25 mg epicatechin/day or a placebo in random order for 14 consecutive days (with a 2 wk washout) [99] resulted in no significant changes in blood pressure.

Among other food sources of catechins, in a placebo-controlled, repeated-dose, double-blind crossover trial, 57 volunteers with borderline hypertension (130–139/85–89 mmHg) or unmedicated mild hypertension (140–165/90–95 mmHg) were allocated to receive apple polyphenol extract rich in epicatechin and flavan-3-ol oligomers (100 mg of epicatechin) or a placebo followed by crossover. The results showed that treatment led to a decrease in DBP by −3.3 mmHg (95% CI: −5.6, −0.9, *p* = 0.008), and an acute improvement in the mean change in maximum FMD% at the first visit of 1.16 (95% CI: 0.04, 2.28 *p* = 0.04), in the last visit of 1.37 (95% CI: 0.22, 2.52, *p* = 0.02), and for both visits combined, 1.29 (95% CI: 0.40, 2.18, *p* < 0.01), although the statistical significance was lost when compared with the placebo. Moreover, the study did not identify any long-term change in either FMD% nor endothelium-independent, nitrate-mediated vasodilatation (NMD) [98].

## 4. Mechanisms of Action of Flavan-3-ols on Vascular Health

### 4.1. Antioxidant Activity

Cardiovascular disease is associated with impairment in endothelial functions and the assessment of a low-grade inflammatory state resulting in an increased production of cytokines [100,101]. The basic inflammatory process may start following a cellular insult due to pro-oxidant stimuli by reactive oxygen species (ROS) (abnormally generated as a by-product of mitochondrial metabolism or by specific enzymes) that exceeds endogenous antioxidant capacity and leads to cell death [102]. The main beneficial effects of flavan-3-ols were attributed to their ROS scavenger activity which is exerted through (1) a direct or (2) an indirect mechanism [103] through the reduction in oxidative damage and lipid peroxidation in vascular smooth muscle cells [104]. The first one consists of the capability of flavan-3-ols to donate an electron and stop ROS production [105,106]. Their antioxidant activity is also ascribed to their ability to chelate metal ions implicated in radical production [107]. Otherwise, the indirect mechanism consists of the ability to regulate the expression of different enzymes involved in ROS scavenging including catalase (CAT), superoxide dismutase (SOD), and glutathione peroxidase (GSH) [108,109,110].

### 4.2. Anti-Inflammatory Regulation

Although the beneficial effect of (poly)phenols on the cardiovascular system have been generally attributed to their antioxidant activity [111], recent knowledge has changed this point of view [112]. Indeed, it is increasingly becoming evident that the protective function of polyphenols may occur through the activation of cellular signaling and the regulation of gene expression [112]. A bioinformatic analysis conducted on nutrigenomic data was performed to investigate the influence of flavan-3-ols in the modulation of gene expression involved in cardiometabolic health [113]. Results identified many genes modulated by flavan-3-ols, not only in endothelial cells and smooth muscle cells but also in hepatocytes, adipocytes, and immune cells that are all involved in the development of the pathology. Here, it was observed that flavan-3-ols mainly modulated different genes implicated in the inflammatory response. In particular, flavan-3-ols acted on transcriptional factors, such as endothelial transcription factor GATA-2 (GATA2), the nuclear factor NF-kappa-B p105 subunit (NF-κB1), forkhead box C1 (FOXC1), peroxisome proliferator-activated receptor-gamma (PPARG), and different miRNA such as let-7b-5p, mir-335-5p, mir-16-5por, and mir-26b-5p [113]. In support of these results, a multi-omic study demonstrated that epicatechin-derived microbial metabolites gamma-valerolactones can regulate the expression of different mRNA, miRNA, and proteins, resulting in the modulation of different cellular pathways involved in cell adhesion, signaling pathways, and interactions with the immune system [114]. In particular, miRNA targets have been found to play a key role in the modulation of different pathways such as endothelial cell functions (adherens junction, gap junction, and focal adhesion), cell signaling (phosphatidylinositol 3-kinase (PI3K)/protein kinase B (PKB/AKT), Wnt, forkhead box O (FOXO), mitogen-activated protein kinases (MAPK), and PPAR signaling), or cell metabolism [114]. Finally, cocoa flavan-3-ols metabolites are able to increase *ApoAI* expression through the involvement of Sites A and B of the *ApoAI* promoter, the major component of HDL which exerts its antiatherogenic properties through the transport of cholesterol to the liver [115].

### 4.3. Flavan-3-ol Microbial-Derived Metabolites and Cardiovascular Health

Flavan-3-ols and proanthocyanidins maintain relative stability during gastric transit [116]. In the small intestine, flavan-3-ol monomers are rapidly converted to generate phase II sulfated, methylated, and glucuronidated derivatives, by the action of sulfotransferases, catechol-*O*-methyltransferases, and uridine-5′-diphosphate-glucuronosyl-transferases, respectively [117]. However, most of the ingested flavan-3-ols are not absorbed in the small intestine, reaching the large intestine and metabolized by the resident microbiota to produce two of their major microbial-derived metabolites, namely the hydroxy-phenyl-gamma-valerolactone (PVLs) and their derived hydroxy-phenylvaleric acids (PVAs), which account for 42% of plasma metabolites following flavan-3-ol intake [117]. Other non-specific flavan-3-ol metabolites include vanillic acid, homovanillic acid, hippuric acid, or p-coumaric acid [118]. Interestingly, unlike the original monomeric molecules, colonic metabolites are more available in the human organism [119]. However, the inter-individual differences in microbial composition, influenced by age, sex, and diet, correlate with changes in the ability to metabolize (poly)phenols [120,121]. Information on specific bacteria strains and the enzymes involved in flavan-3-ol metabolism to PVLs/PVAs is very limited. *Flavonifractor plautii* can convert 1-(3′,4′-dihydroxyphenyl)-3-(2″,4″,6″-trihydroxyphenyl)-propan-2-ol to 5-(3′,4′-dihydroxyphenyl)-gamma-valerolactone and 4-hydroxy-5-(dihydroxyphenyl)valeric acid [122]. However, another study investigating the interindividual difference in flavan-3-ol metabolism demonstrated that the production of dihydroxyphenyl-gamma-valerolactone conjugates was influenced by interindividual microbial composition with a positive correlation with Clostridia and Actinobacteria class, including the species *F. plautii*, Clostridium leptum, *Sporobacter termitidis*, Ruminococcus bromii, and *Eubacterium ramulus*, and the genus *Propionibacterium* [123]. Similar results have been reported in another study in which high levels of PVLs and PVAs in the plasma and urine were correlated with *Dialister*, *Prevotella* and *Escherichia*/*Shigella*, respectively, while a negative correlation was observed with bacteria *Anaerostipes*, *Turicibacter*, *Blautia,* and *Lachnospiraceae* in the levels of these metabolites in the plasma and urine, respectively [124].

Furthermore, an in vitro study demonstrated the involvement of *Akkermansia muciniphila* in the degradation of EGCG. However, it was demonstrated that *A. muciniphila* did not metabolize EGCG alone but in the presence of either mucin or glucose substrate [125] and converted EGCG into gallic acid, epigallocatechin, and (−)-epicatechin [125], promoting its own grown as demonstrated by many other works [126,127].

**Figure 1:**
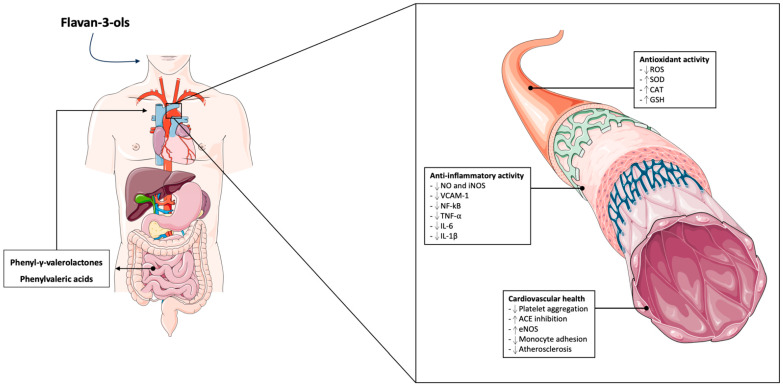
A schematic overview of the primary activities of microbial-derived metabolite flavan-3-ols. ACE (angiotensin-converting enzyme); CAT (catalase); eNOS (endothelial nitric oxide synthase); GSH (glutathione peroxidase); IL-1β (interleukin-1β); IL-6 (interleukin-6); iNOS (inducible nitric oxide synthase); NF-κB (nuclear factor-kappa B); NO (nitric oxide); ROS (reactive oxygen species); SOD (superoxide dismutase); TNF-α (tumor necrosis factor-alpha).

The cardioprotective properties attributed to flavan-3-ols could be associated with their microbial-derived metabolites PVLs and PVAs through their anti-inflammatory activity, hypotensive properties, and ability to reduce the adhesion of monocyte to endothelial cells, preventing atherosclerosis development as represented in Figure 1. 5-(3′,4′-dihydroxyphenyl)-gamma-valerolactone has been tested for its anti-inflammatory properties, showing the capacity to inhibit the expression of iNOS [128] which increased activity has been correlated with heart failure [129]. Moreover, two microbial-derived metabolites 5-(3,4,5-trihydroxyphenyl)-gamma-valerolactone and 5-(3,5-dihydroxyphenyl)-gamma-valerolactone showed hypotensive properties in an in vivo study conducted on rats [130]. Hydroxyphenyl valeric acids, also derived by flavan-3-ols metabolism, demonstrated a strong inhibitory activity of angiotensin I-converting enzymes (ACEs) [130]. The cardioprotective effect of the microbial-derived metabolite 5-(3′,4′-dihydroxyphenyl)-gamma-valerolactone has been demonstrated by another in vitro study, demonstrating that the treatment prevents monocyte adhesion to endothelial cells through downregulation of VCAM-1 in tumor necrosis factor-alpha (TNF-alpha)-treated cells [131]. This mechanism could be attributed to a downregulation of phosphorylation and the activation of NF-κB [131]. Microbial catabolism of flavan-3-ols leads also to the production of phenolic acids [132] showing anti-inflammatory, antioxidant, and cardioprotective effects. Among catabolic metabolites derived by flavan-3-ols, *trans*-caffeic acid and 3,4-dihydroxyphenylpropionic acid (3,4-DHPP) inhibited NO production in lipopolysaccharides (LPS)-treated RAW264.7 cells [133]. Furthermore, in vitro treatment with benzoic acid-sulfate, vanillic acid-glucuronide, and protocatechuic acid-3-sulfate leads to a reduction in TNF-alpha secretion induced by LPS stimulation [134]. Similar results demonstrated a dose-dependent response to protocatechuic acid treatment, leading to a reduction in TNF-alpha, interleukin 6 (IL-6), IL-1 beta, and prostaglandin E2 production as well as a reduced expression of toll-like receptor 4 (TLR4), activation of NF-κB, and MAPKs [135]. In another study, protocatechuic acid used at a concentration between 5 and 25 uM showed a significant reduction in platelet aggregation which was mediated by blocking the interaction between the von Willebrand factor (vWF) and the platelet receptor glycoprotein Ib [136]. Furthermore, it was demonstrated that protocatechuic acid administration to type 1 diabetic rats decreased plasma glycated hemoglobin (HbA1c) and cardiac malondialdehyde (MDA) levels, indicating an improvement of cardiac mitochondrial function, and increased anti-apoptotic B-cell lymphoma 2 (BCL2) expression [137]. Protocatechuic acid also showed hypoglycemic and insulin-sensitizing activity, exerted via the stimulation of the poly (ADP-ribose) polymerase (PARP)/protein kinase C (PKC)/NF-κB pathway in myocardial tissue [138]. 3-hydroxyphenylpropionic acid (3-HPP) was also reported to have beneficial effects on the endothelium through the increased phosphorylation of eNOS and Akt, a pathway that has been greatly demonstrated to be protective for endothelial function [139].

## 5. Future Perspectives

This review summarized the main results from clinical studies exploring the effects of flavan-3-ols on vascular outcomes. Although most trials reported significant effects on vascular health, including reductions in blood pressure and improved endothelial function, not all reported results in the literature are univocal. Thus, the evidence from reviewed studies should be considered in light of some limitations. First, the heterogeneity among the reviewed trials related to differences in the health status of the target population and characteristics of exposure, including differences in the doses, supplement composition, and trial duration, may at least partially explain the discrepancies among the explored studies. Second, the limitations comprise the potential interactions, including accumulating, synergistic, and antagonistic effects, with other supplement components as well as food matrix components. Finally, the variations in the actual exposure to flavan-3-ol metabolites related to the differences in gut microbiota composition cannot be ruled out. Therefore, future clinical studies exploring variables that may influence the intra- and inter-individual variations in the bioavailability of flavan-3-ols to better elucidate the role of flavan-3-ols and their metabolites in vascular functioning are warranted.

## 6. Conclusions

Clinical trials demonstrated that supplementation with flavan-3-ols, primarily derived from cocoa products, exhibits significant effects in reducing blood pressure and ameliorating endothelial function. Studies focusing on catechins have shown enhanced outcomes when the supplementation occurred through green tea drinks in healthy individuals. Moreover, quantitative analyses support the efficacy of green tea supplementation, including extracts, in reducing blood pressure, particularly in hypertensive individuals or in those at high risk of CVD. From a mechanistic point of view, emerging evidence suggests that microbial metabolites may play a role in the observed effects. Their function extends beyond the previous belief of ROS scavenging activity and encompasses a direct impact on gene expression and protein function. However, more studies are necessary to delve deeper into the role of the microbiota and the inter-individual variations in gut microbiota composition, aiming to gain a comprehensive understanding of how differences in microbiota-derived metabolites may influence cardiovascular risk.

## Figures and Tables

**Table 1:** Main characteristics of randomized clinical trials concerning flavan-3-ols supplementation and cardiovascular risk.

Author, Year, Country	Study Design	Participants (Mean Age)	Duration	Treatment	Polyphenol Constituent (Daily Intake)	Comparison	Main Findings
Murphy, 2003, Australia [38]	Double-blind, placebo-controlled	32 healthy subjects (treatment group: 40 y; placebo group: 47 y)	28 d	Tablets containing flavanols and procyanidins	234 mg flavanols and procyanidins	Placebo tablets (≤6 mg flavanols and procyanidins)	No divergences were seen between groups after treatment.
Engler, 2004, USA [39]	Double-blind, placebo-controlled	21 healthy volunteers (38 y)	2 wk	Dark chocolate bars	213 mg total procyanidins and 46 mg epicatechin	Placebo dark chocolate bars (traces of procyanidins and epicatechin)	High-flavonoid chocolate intake was associated with a significant amelioration of FMD (*p* = 0.024).
Fraga, 2005, Argentina [40]	Crossover, placebo-controlled	28 healthy participants (19 y)	2 × 14 d	Flavanol-containing milk chocolate	168 mg flavanols (39 mg epicatechin and catechin and 126 mg of procyanidins)	Cocoa butter chocolate (<5 mg/d of flavanols)	FCMC consumption was associated with a significant decrease in DBP (*p* = 0.01) and the mean BP (*p* = 0.008).
Grassi, 2005, Italy [41]	Crossover, placebo-controlled	15 normotensive control (34 y) and 20 never-treated, grade I essential hypertensive individuals (43 y)	2 × 15 d (7 d washout)	Dark chocolate bars	88 mg flavanols (21.91 mg catechin, 65.97 mg epicatechin, 0.59 mg quercetin, 0.03 mg kaempferol, and 0.31 mg isorhamnetin)	Flavanol-free white chocolate bars	SBP and DBP significantly decreased after dark chocolate consumption compared to white chocolate and baseline values (*p* < 0.0001). FMD increased after dark chocolate consumption but not after white chocolate consumption (*p* < 0.0001).
Wang-Polagruto, 2006, USA [42]	Double-blind, placebo-controlled	32 postmenopausal hypercholesterolemic women (high-flavanol group: 57 y; low-flavanol group: 55 y)	6 wk	High-flavanol cocoa beverage	446 mg flavanols	Placebo cocoa beverage (43 mg flavanols)	Low-flavanol consumption led to a significant decrease in SBP and DBP (*p* < 0.05). High-flavanol treatment led to an increase in brachial artery hyperemic blood flow after (*p* < 0.05) compared to the baseline.
Baba, 2007, Japan [43]	Double-blind, placebo-controlled	25 healthy male subjects (38 y)	12 wk	Cocoa powder + sugar	98.02 mg epicatechins, 35.1 mg catechins, 41.08 procyanidin B2, and 24.9 mg procyanidin C1	Sugar	No significant results were found in SBP and DBP.
Taubert, 2007, Germany [44]	Investigator-blinded, placebo-controlled	44 participants (64 y)	18 wk	Dark chocolate	30 mg polyphenols (catechin 1.7 mg, epicatechin 5.1 mg, epicatechin-gallate 0.3 mg, procyanidin dimer 6.8 mg, procyanidin dimer-gallate 1.8 mg, procyanidin tetramer 3.7 mg, procyanidin pentamer 2.6 mg, and flavonols < 0.05 mg)	Polyphenol-free white chocolate	Dark chocolate consumption reduced SBP and DBP (*p* < 0.001).
Abdullah A, 2008, Saudi Arabia [45]	Placebo-controlled	89 healthy female (21 y)	15 d	(i) Dark chocolate	1.89 mg gallic acid, 1.08 mg ECG, 3.99 mg catechin, caffeine 16.99 mg, 4.01 mg EC, epigallocatechin 3-gallate 1.40 mg, and epicatechin 3-gallate 0.14 mg	(ii) White chocolate or no chocolate; (iii) no chocolate	Dark chocolate intake was associated with decreased SBP and DBP (*p* < 0.05).
Crews, 2008, USA [46]	Double-blind, placebo-controlled	90 healthy participants (≥60 y)	6 wk	Dark chocolate bar and artificially sweetened cocoa beverage	754.71 mg total proanthocyanidins/g	Placebo bar (0.20 mg/g proanthocyanin) and beverage (40.87 mg/g proanthocyanin)	The dark chocolate and cocoa groups displayed an increased pulse rate at the midpoint and at the end of treatment (*p* = 0.007).
Davison, 2008, Australia [47]	Double-blind, placebo-controlled	49 participants (41 y)	12 wk	High-flavanol cocoa drink	902 mg flavanols	Low-flavanol cocoa drink (36 mg/d flavanols)	High-flavanol cocoa intake increased FMD (*p* < 0.01) and reduced DBP and mean arterial BP (*p* < 0.05).
Grassi, 2008, Italy [48]	Placebo-controlled, crossover	19 essential hypertension participants (44 y)	2 × 15 d (7 d washout)	Flavanol-rich dark chocolate	1008 mg total phenols (110.9 mg EC, 36.12 mg catechin, 2.5 mg quercetin, 0.03 mg kaempferol, and 0.2 mg isorhamnetin)	Flavanol-free white chocolate	Flavanol-rich chocolate reduced the SBP, DBP and ambulatory pressure which was inversely correlated with an increase in FMD with FRDC (*p* < 0.0001).
Muniyappa, 2008, USA [49]	Double-blind, placebo-controlled, crossover	20 participants with mild-to-moderate hypertension (43 y)	2 × 2 wk (1 wk washout)	Flavanol-rich cocoa drink	902 mg cocoa polyphenols (174 mg EC, 62 mg catechin, and 676 mg procyanidins)	Flavanol-poor placebo (28 mg cocoa polyphenols: 2 mg epicatechin, 8 mg catechin, and 16 mg procyanidins)	Cocoa treatment for 2 wk was able to increase insulin-stimulated brachial arterial dilatation compared to placebo (*p* = 0.028).
Shiina, 2009, Japan [50]	Single-blind, placebo-controlled	39 healthy men (29 y)	2 wk	Flavonoid-rich dark chocolate	Catechin, epicatechin, and procyanidin (550 mg)	White chocolate	Dark chocolate consumption was associated with increased change in CFVR compared to control group (*p* < 0.01).
Heiss, 2010, USA [51]	Double-blind, placebo-controlled, crossover	16 participants CAD (64 y)	2 × 30 d (1 wk washout)	Cocoa drink	750 mg flavanols, monomers–decamers, 130 mg monomers, 118 mg EC, 12 mg catechin, 106 mg dimers, and 516 mg trimers–decamers	Placebo cocoa drink (18 mg flavanols, monomers–decamers, 6 mg monomers, 2 mg EC, 4 mg catechin, 4 mg dimers, and 6 mg trimers–decamers)	High-flavanol intervention led to a significant increase in FMD (*p* < 0.05) and a decrease in SBP (*p* = 0.013).
Njike, 2011, USA [52]	Double-blind, placebo-controlled crossover	37 healthy participants (52 y)	2 × 6 wk (4 wk washout)	(i) Sugar-free cocoa beverage; (ii) sugar-sweetened cocoa beverage	21 mg catechin, 48 mg epicatechin, 92 mg procyanidin dimer, 98 mg procyanidin trimer, 31 mg procyanidin tetramer, 55 mg procyanidin pentamer and hexamer, and 805 mg total procyanidin	Sugar-sweetened cocoa-free beverage	Cocoa ingestion improved FMD relative to the control group (*p* < 0.01).
Almoosawi, 2012, UK [53]	Single-blind placebo-controlled, crossover	42 women (21 normal BMI, 13 overweight, and 8 obese)	2 × 4 wk (2 wk washout)	Dark chocolate	500 mg total polyphenols, 18.99 mg epicatechin and catechin	Placebo dark chocolate	Decrease in SBP (*p* = 0.007) and DBP (*p* = 0.003) after dark chocolate intake.
Desideri, 2012, Italy [54]	Double-blind	90 elderly individuals	8 wk	(i) High-flavanol cocoa drink; (ii) intermediate-flavanol cocoa drink	(i) 990 mg flavanols; (ii) 520 mg flavanols	Low flavanols drink (45 mg flavanols)	High and intermediate flavanol intake was associated with reduced BP after 8 wk of treatment (*p* < 0.0001) and compared to the low group (*p* < 0.05).
Flammer, 2012, Switzerland [55]	Double-blind, placebo-controlled	20 participants with congestive heart failure (64 y)	4 wk	Flavanol-rich chocolate	10.8 mg catechin, 36 mg epicatechin	Placebo chocolate	Flavanol-rich chocolate administration led to significant increase in FMD (*p* = 0.045). No changes were observed for BP and HR.
Mogollon, 2013, Canada [56]	Double-blind, placebo-controlled	42 healthy, pregnant women (29 y)	12 wk	High-flavanol dark chocolate	400 mg total flavanols, 64 mg total catechin and epicatechin	Low-flavanol chocolate	Results showed no differences.
Neufingerl, 2013, The Netherlands [57]	Double-blind, placebo-controlled	143 healthy participants (theobromine + cocoa group: 55 y; theobromine group: 53 y; cocoa group: 55 y; placebo group: 55 y)	4 wk	(i) Cocoa drink; (ii) theobromine drink; (iii) theobromine + cocoa drink	(i) 325 mg flavanols, 150 mg theobromine; (ii) 850 mg theobromine; (iii) 325 mg flavanols, 1000 mg theobromine	Placebo drink	No changes were reported in SBP, DBP, and HR among the groups.
Esser, 2014, UK [58]	Double-blind, placebo-controlled, crossover	41 overweight, middle-aged men (57 y)	4 wk (4 wk washout)	High-flavanol chocolate	1078 mg flavanols, 349 mg epicatechins	Normal flavanol chocolate	An increase in FMD by 1% and a decrease in the AIX independent of the type of chocolate consumed.
Ibero-Baraibar, 2014, Spain [59]	Double-blind, placebo-controlled	47 healthy participants with BMI of 30.59 ± 2.33 kg/m^2^ (57 y)	4 wk	Cocoa extract	414.26 mg total flavanols, 153.44 mg epicatechin, 14.56 mg catechin, 99.40 mg dimer B2, 13.44 mg dimer B1, and 133.53 mg oligomeric procyanidins	Control meal	Both groups showed an improvement in SBP and DBP after 4 weeks of intervention.
Nickols-Richardson, 2014, USA [60]	Placebo-controlled	51 overweight/obese postmenopausal women (35 y)	18 wk	Sugar-free natural cocoa beverage + chocolate snack	270 mg flavanols	Non-cocoa beverage and non-cocoa snack	Both groups observed a significant reduction in SBP and DBP independent of intervention.
Sarriá, 2014, Spain [61]	Placebo-controlled, crossover	24 normocholesterolemic (M: 28 y; F: 26 y) and 20 moderately hypercholesterolemic (M: 35 y; F: 25 y) volunteers	2 × 2 wk	Soluble cocoa powder + milk	416.4 mg polyphenols, 44.1 mg flavanols, 9.3 mg epicatechins, 18 mg catechins, and 16.5 mg procyanidin B2	Milk	No significant changes were observed in SBP, DBP, and HR between groups.
West, 2014, USA [62]	Placebo-controlled, crossover	30 middle-aged, overweight, and moderately obese adults (51 y)	2 × 4 wk (2 wk washout)	Dark chocolate + sugar-free cocoa beverage	814 mg total flavanols	Low-flavanol chocolate bar and cocoa-free beverage with no added sugar (3 mg/d total flavanols)	Increase in brachial artery diameter before hyperemia (*p* = 0.001) and at peak dilation following cuff release (*p* = 0.0001). Resting (*p* = 0.04) and peak (*p* = 0.03) hyperemic blood flow increased after treatment. Substantial decreases in the AIX were observed only in women (*p* = 0.01).
Heiss, 2015, Germany. [63]	Double-blind, placebo-controlled	42 healthy participants (22 young (26 y); 20 elderly (60 y)	14 d	CF drink	900 mg flavanols, 146 mg monomers, 128 mg (-)-epicatechin, 14 mg (-)-catechin, 4 mg (+)-catechin, and 754 mg dimers–decamers	CF free	Improvement in FMD (*p* < 0.001), reduced PWV and lowered total peripheral resistance, and increased arteriolar and microvascular vasodilatory capacity, and central DBP in both groups. In elderly group, CF decreased the aortic AIX and office peripheral SBP (*p* < 0.05).
Koli, 2015, Finland [64]	Crossover	22 participants with mild hypertension (45 y)	2 × 8 wk (4 wk washout)	Dark chocolate	602.7 mg flavanols	Snack restriction	No changes were observed after dark chocolate intake.
Massee, 2015, Australia [65]	Double-blind, placebo-controlled	38 healthy participants (24 y)	4 wk	Cocoa tablet	250 mg catechin	Placebo tablets (inert cellulose powder)	No differences were observed compared to baseline values.
Mastroiacovo, 2015, Italy [66]	Double-blind, placebo-controlled	90 elderly participants (69 y)	8 wk	(i) High flavanol drink; (ii) intermediate-flavanol drink	(i) 993 mg total flavanols, 185 mg epicatechin, 62 mg catechin, 182 mg dimers, 141 mg trimers, 126 mg tetramers, 297 mg pentamers–decamers; (ii) 520 mg total flavanols, 95 mg epicatechin, 35 mg catechin, 96 mg dimers, 72 mg trimers, 64 mg tetramers, and 158 mg pentamers–decamers	Low flavanol drink containing 48 mg total flavanols, 5 mg epicatechin, 8 mg catechin, 10 mg dimers, 4 mg trimers, 2 mg tetramers, and 17 mg pentamers–decamers	SBP was significantly reduced in HF and IF after treatment (*p* < 0.0001) and compared to the LF group (*p* < 0.0001).
Ottaviani, 2015, USA [67]	Double-blind, placebo-controlled	59 healthy adults (41 y)	12 wk	Cocoa extract capsules	Up to 2000 mg cocoa flavanols (248 mg flavanol monomers, 1752 mg procyanidins)	Placebo capsules, cocoa-flavanol-free	Treatment was not associated with significant changes in BP or platelet function.
Rassaf, 2016, Germany [68]	Double-blind, placebo-controlled	49 participants with ESRD (65 y)	30 days	Cocola-flavanol-rich beverage	900 mg total cocoa flavanols	Placebo beverage	A significant increase in FMD (*p* < 0.001), a reduction in DBP (*p* = 0.03), and an increase in HR (*p* = 0.01) compared to the placebo group.
Rull, 2015, UK [69]	Double-blind placebo-controlled, crossover	32 healthy participants (55 years)	2 × 6 wk	High-flavanol dark chocolate	1064 mg total flavanols, 223 mg monomers, 192 mg procyanidin dimers, and 649 mg procyanidin trimers to decamers	Low-flavanol dark chocolate (88 mg total flavanols, 23 mg monomers, 24 mg procyanidin dimers, and 41 mg procyanidin trimers to decamers)	The LFDC group showed an increase in 24 h (*p* = 0.008) and daily HR (*p* = 0.001) compared to pre-study values.
Sansone, 2015, Germany [70]	Double-blind, placebo-controlled	100 healthy individuals (47 y)	1 mo	Cocoa-flavanol drink	900 mg total flavanols, 146 mg monomers, 128 mg (-)-epicatechin, 14 mg (-)-catechin, 4 mg (+)-catechin, and 754 mg dimers–decamers	Cocoa-flavanol-free drink	The CF group showed an increase in FMD and a decrease in office and central SBP and DBP. PWV and the AIX decreased in the CF group compared to the control group.
Njike, 2016, USA [71]	Double-blind, placebo-controlled	101 adults with stage 1 hypertension (140–159/90–99 mmHg) on no more than one BP medication (53 y)	2 × 8 wk (4 wk washout)	(i) 10 g cocoa powder; (ii) 5 g cocoa powder	(i) 261.8 mg, flavanols, 45.8 mg epicatechin; (ii) 130.9 mg flavonols, 22.9 mg epicatechin	Placebo chocolate	Treatment reduced BP after subgroup analysis (24 h SBP, *p* = 0.038, 24 h DBP, *p* = 0.023). It reduced BP (*p* = 0.009) and improved endothelial function (*p* = 0.031) in participants on beta blockers. Participants on diuretics daily consumption showed significant increase in 24 h ambulatory SBP (*p* = 0.022).
Garcia-Yu, 2020, Spain [72]	Controlled	137 postmenopausal women (intervention group 57 y; control group 57 y)	6 mo	Chocolate (99% cocoa)	0.58 mg protocatechuic acid, 1.76 mg procyanidin dimer (B3), 10.4 mg catechin, 14.4 mg procyanidin dimer (B2), 26.1 mg epicatechin,8.53 mg procyanidin trimer (C1), 3.54 mg procyanidin A hexoside, 0.02 mg quercetin glucoside, and 0.03 mg quercetin arabinoside	No intervention	No notable changes were observed between groups for SBP, DBP, or brachial–ankle PWV. For PP, in contrast to the increase observed in the control group, there was a decrease in the intervention group (*p* = 0.048).

Abbreviations: AIX (augmentation index); BP (blood pressure); CAD (coronary heart disease); CFVR (coronary flow velocity reserve); d (day); DBP (diastolic blood pressure); EC (epicatechin); ECG (epicatechin gallate); ESRD (end-stage renal disease); F (female); FMD (flow-mediated dilation); FRDC (flavanol-rich dark chocolate); HF (high flavanol); HR (heart rate); IF (intermediate flavanol); LF (low flavanol); LFDC (low-flavanol dark chocolate); M (male); mo (month); PWV (pulse wave velocity); SBP (systolic blood pressure); wk (week); y (year).

## Abbreviations

3,4-DHPP (3,4-dihydroxyphenylpropionic acid); 3-HPP (3-hydroxyphenylpropionic); ACE (angiotensin-converting enzyme); ADP (adenosine diphosphate); AIX (augmentation index); AKT (protein kinase B); (ApoAI) apolipoprotein AI; BCL2 (B-cell lymphoma 2); BMI (body mass index); BP (blood pressure); bpm (beats per minute); CAT (catalase); CFVR (flow velocity reserve); CI (confidence interval); CVD (cardiovascular disease); DBP (diastolic blood pressure); EGCG (epigallo-catechin-3-gallate); eNOS (endothelial nitric oxide synthase); ERK1/2 (extracellular signal-regulated kinase); FMD (flow-mediated dilation); FOXC1 (forkhead box C1); Foxo (forkhead box o); GSH (glutathione peroxidase); HbA1c (Glycated hemoglobin); HDL (high-density lipoprotein); HR (heart rate); ICAM-1 (intercellular adhesion molecule-1); IL (interleukin); iNOS (inducible nitric oxide synthase); LPS (lipopolysaccharides); MAPK (mitogen-activated protein kinase); MDA (malondialdehyde); NF-κB (nuclear factor-kappa B); NMD (nitrate-mediated vasodilatation); NO (nitric oxide); PI3K-Akt (phosphatidylinositol 3-kinase-Akt); PKC (protein kinase C); PARP (poly (ADP-ribose) polymerase); PPAR (peroxisome proliferator-activated receptor); PPARG (peroxisome proliferator-activated receptor gamma); PVAs (hydroxy-phenylvaleric acids); PVLs (hydroxy-phenyl-gamma-valerolactone); PWV (pulse wave velocity); RCT (randomized clinical trial); ROS (reactive oxygen species); SBP (systolic blood pressure); sICAM1 (soluble intercellular cell adhesion molecule-1); sICAM3 (soluble intercellular cell adhesion molecule-3); SOD (superoxide dismutase); TLR4 (toll-like receptor 4); TNF-alpha (tumor necrosis factor-alpha); VCAM-1 (vascular cell adhesion protein-1); vWF (von Willebrand factor); wk (week); wnt (wingless/integrated); y (year).
